# Unusual context of *CENPJ* variants and primary microcephaly: compound heterozygosity and nonconsanguinity in an Argentinian patient

**DOI:** 10.1038/s41439-020-0105-3

**Published:** 2020-06-08

**Authors:** Anna M. Cueto-González, Mónica Fernández-Cancio, Paula Fernández-Alvarez, Elena García-Arumí, Eduardo F. Tizzano

**Affiliations:** 1 Department of Clinical and Molecular Genetics, Vall d’Hebron Barcelona Hospital Campus, Barcelona, Spain; 2 Medicine Genetics Group, Vall d´Hebron Research Institute (VHIR), Vall d’Hebron Barcelona Hospital Campus, Autonomous University of Barcelona, Barcelona, Spain; 3 European Reference Network Craniofacial Anomalies and ENT disorders (ERN CRANIO)(member) and ERN ITHACA (affiliated), Barcelona, Spain; 4 Pediatric Endocrinology Unit, Vall d´Hebron Research Institute (VHIR), Vall d’Hebron Barcelona Hospital Campus, Autonomous University of Barcelona, Barcelona, Spain; 5 Centro de Investigación Biomédica en Red de Enfermedades Raras (CIBERER), Instituto de Salud Carlos III, Barcelona, Spain; 6 Neuromuscular and Mitochondrial Pathology Group, Vall d’Hebron Research Institute (VHIR), Vall d’Hebron Barcelona Hospital Campus, Autonomous University of Barcelona, Barcelona, Spain

**Keywords:** Genetics research, Neurological disorders

## Abstract

Primary microcephaly (MCPH) is a genetically heterogeneous disorder showing an autosomal recessive mode of inheritance. Patients with MCPH present head circumference values two or three standard deviations (SDs) significantly below the mean for age- and sex-matched populations. MCPH is associated with a nonprogressive mild to severe intellectual disability, with normal brain structure in most patients, or with a small brain and gyri without visceral malformations. We present the case of an adult patient born from Argentinian nonconsanguineous healthy parents. He had a head circumference >5 SD below the mean, cerebral neuroimaging showing hypoplasia of the corpus callosum, bilateral migration disorder with heterotopia of the sylvian fissure and colpocephaly. The patient was compound heterozygous for pathogenic variants in the *CENPJ* gene (c.289dupA inherited from his mother and c.1132 C > T inherited from his father). Our patient represents an uncommon situation for the usual known context of *CENPJ* and MCPH, including family origin (Argentinian), pedigree (nonconsanguineous), and genotype (a compound heterozygous case with two variants predicting a truncated protein). Next-generation sequencing studies applied in a broader spectrum of clinical presentations of MCPH syndromes may discover additional similar patients and families.

Primary microcephaly (MCPH) is a genetically heterogeneous congenital disorder of brain development. Most cases of MCPH show an autosomal recessive mode of inheritance. Affected patients present head circumference values equal to or greater than two to three standard deviations (SDs) below the age- and sex-matched population mean. MCPH is associated with a nonprogressive mild to severe intellectual disability and the absence of visceral malformations^1,2^. There are some discrepancies with the parameters to define and consider MCPH in a patient. A proportion of papers define MCPH as having a head circumference value ≥2 SDs below the population mean (~2% of the general population is included in this definition), and others consider a value that is ≥3 SDs below the mean (0.1% of the population is included)^1^. MCPH is caused by mutations in at least 25 different genes involved in DNA damage repair, orienting the mitotic spindle, or in cell division control^3–6^ (Table 1).

**Table 1:** Update of the 25 subtypes of primary autosomal recessive microcephaly and related causative genes; MCPH = primary microcephaly (compiled from ref. ^5^).

MCPH subtype	OMIM phenotype number	Gene	OMIM genotype number
MCPH1	251200	*MCPH1*	607117
MCPH2	604317	*WDR62*	613583
MCPH3	604804	*CDK5RAP2*	608201
MCPH4	604321	*KNL1*	609173
MCPH5	608716	*ASPM*	605481
MCPH6	608393	*CENPJ*	609279
MCPH7	612703	*STIL*	181590
MCPH8	614673	*CEP135*	611423
MCPH9	614852	*CEP152*	613529
MCPH10	615095	*ZNF335*	610827
MCPH11	615414	*PHC1*	602978
MCPH12	616080	*CDK6*	603368
MCPH13	616051	*CENPE*	117143
MCPH14	616402	*SASS6*	609321
MCPH15	616486	*MFSD2A*	614397
MCPH16	616681	*ANKLE2*	616062
MCPH17	617090	*CIT*	605629
MCPH18	617520	*WDFY3*	617485
MCPH19	617800	*COPB2*	606990
MCPH20	617914	*KIF14*	611279
MCPH21	617983	*NCAPD2*	615638
MCPH22	617984	*NCAPD3*	609276
MCPH23	617985	*NCAPH*	602332
MCPH24	618179	*NUP37*	609264
MCPH25	618351	*MAP11*	618350

We present the case of an adult patient born in Argentina from Argentinian nonconsanguineous parents. Next-generation sequencing (NGS) of MCPH genes revealed a compound heterozygous genotype with two pathogenic variants in *CENPJ* (OMIM 609279).

Our patient is, at present, 20-years old, and he has been followed-up in several Argentinian and Spanish hospitals because of his clinical manifestations. He is the only son of an Argentinian healthy nonconsanguineous couple (age of parents at birth, mother 19-years old and father 26-years old). No other relevant familial pathological cases were reported. His mother had only one echography at 3 months of pregnancy, and normal results were reported. No environmental factors, such as infections or drugs, were noted during pregnancy. The patient’s birth weight was 2.8 kg (−1.38 SD), birth length was 48 cm (−1.44 SD), and head circumference was 30.5 cm (−3.05 SD). Psychomotor delay began during infancy, and progressively, moderate to severe intellectual disability was noted, together with hyperactivity. The present physical examination shows a head circumference of 48 cm (−5.42 SD) with height and weight in the third percentile. His height (167 cm, −1.63 SD) is lower than his mean parental height (179 ± 5 cm, +0.24 SD; mother is 170 cm and father is 175 cm). Severe MCPH, a sloping forehead, a long nasal columella, markedly crowded teeth, and mild pectus excavatum were present. No other relevant phenotypic characteristics were noted. The parents (legal guardians) gave written informed consent (approved by Vall d´Hebron Ethical Committee) for biochemical and molecular studies, but refused to permit pictures to be taken of the patient. A number of complementary tests were performed, including karyotype (46,XY) and comparative genome hybridization array (reported as normal); metabolic screening (normal); the exclusion of maternal phenylketonuria; echocardiogram (normal); and cerebral magnetic resonance imaging (performed at 9 years, reporting hypoplasia of the corpus callosum, bilateral migration disorder with heterotopia of the sylvian fissure and colpocephaly).

Genomic DNA of the patient and his parents was extracted from peripheral blood mononuclear cells using standard procedures. For library preparation, a commercially available targeted resequencing kit, the TruSight One (TSO) Sequencing Panel, was used, and the DNA samples were sequenced on a MiSeq platform (Illumina, San Diego, CA, USA). The TSO Sequencing Panel kit allows the analysis of 4813 target genes associated with human genetic disorders, including 38 autosomal recessive MCPH genes related to isolated MCPH, and other recessive entities with primary MCPH and other malformations (example: the *TUBGCP6* and *PLK4* genes, which are related to MCPH associated with chorioretinopathy; http://www.illumina.com/products/trusight-one-sequencing-panel.html). All procedures were prepared according to the manufacturers’ instructions. Data were analyzed using MiSeq Reporter software (Illumina) and mapped to the human genome reference sequence (GRCh37, hg19).

For data analysis, we used VariantStudio version 2.2.1 (Illumina) and Integrative Genomics Viewer software version 2.3.57 (Broad Institute, Cambridge, MA, USA). The DNA sample of this patient generated ~9700 variants. Filtering criteria included a coverage of >20 reads, a variant frequency of >20%, and a minor allele frequency of <0.02% in the 1000 Genomes database and the gnomAD browser (http://gnomad.broadinstitute.org; NHLBI Exome Sequencing Project), and selected for variant types, such as missense, nonsense, synonymous, and splice variants. The first analysis filtered for a recessive mode of inheritance in candidate genes for autosomal recessive MCPH. The pathogenicity of variants was ascertained according to the criteria of the American College of Medical Genetics ^7^.

Two pathogenic variants were detected: c.289dupA (p.(Thr97AsnfsTer7); reported by our group in ClinVar: SCV000986655) and c.1132 C > T (p.(Arg378Ter)) in the *CENPJ* gene (NM_018451.4, NP_060921.3; reported by our group in ClinVar: SCV000986656). These variants were confirmed by Sanger sequencing (Fig. 1). His mother is a c.289dupA carrier, and his father is a c.1132 C > T carrier. Both mutations were found in the gnomAD browser with frequencies of 0.014% and 0.012%, respectively, in the Latino population. The two variants were considered pathogenic because both predicted the generation of a truncated protein of 103 and 378 amino acids, respectively, instead of the 1338 amino acids of the wild-type protein. Both variants were considered deleterious by in silico predictors (UMD predictor, PROVEAN, Mutation Taster).

**Fig. 1:**
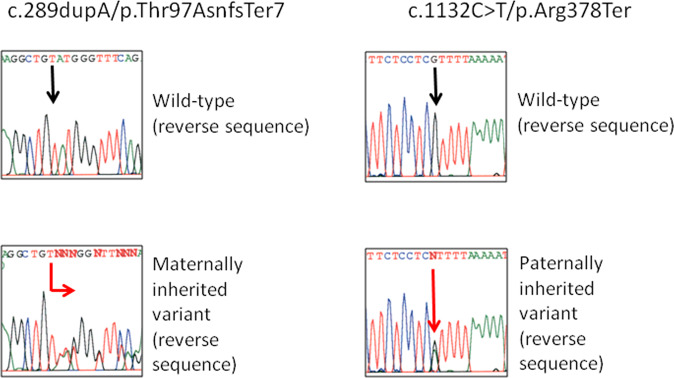
Electropherogram of the two pathogenic variants found in our patient. The upper part shows the wild-type genotype, and the lower part shows the genotype of the patient (reverse sequences).

This is the first report of an MCPH patient who is compound heterozygous for two truncating variants in *CENPJ*. To the best of our knowledge, this is also the first case of Argentinian origin reported, given that these patients are usually more common in other populations with higher inbreeding rates. To date, all the mutations identified in the *CENPJ* gene have been reported in a homozygous state, and the vast majority have been reported in consanguineous couples from Pakistan, Brazil, southern Saudi Arabia, and Turkey (Table 2 compiles families and variants reported so far, including our patient). All these reported variants were novel, with the exception of c.18delC (p.Ser7Profs*2), which was reported to be recurrent in Pakistani patients^8^. *CENPJ* pathogenic variants have been described in patients with autosomal recessive primary MCPH type 6 (OMIM 608393)^9,10^, Seckel syndrome type 4 (OMIM 613676)^11^/primordial dwarfism, and in a patient with arthrogryposis and abnormal scar formation, although this last patient had normal growth parameters, including head circumference^6,12^ (Table 2). Primary MCPH and primordial dwarfism are considered to belong to a continuous spectrum of phenotypic manifestations, without a clear genotype–phenotype correlation^6^; this may explain in our patient the head circumference >3 SD below the population mean, with a height lower than expected according to the height of his parents. However, the height seen in Seckel syndrome/primary dwarfism is usually lower (with intervals between 4 and 12 SDs below the population mean)^2^.

**Table 2:** Compilation of reported cases with microcephaly according to geographic origin, clinical findings, and *CENPJ* genotypes; SD = standard deviation, NA = not available information, and NR = not reported in the patient.

Family origin	Clinical findings			Variant in C*ENPJ*	Number of cases reported/described	Ages reported	Zygosity	Reference
	Reduced head circumference	Intellectual disability	Other features					
Pakistani	Yes	NA	Sloping forehead	c.18delC	3	NA	Homozygous	Bond et al.^9^ (and reference therein, Leal et al., 2003)
				p.(Ser7Profs*2)				
Pakistani	Yes	NA	Sloping forehead	c.18delC	4	NA	Homozygous	
				p.(Ser7Profs*2)				
Brazilian	Below −7 SD	Moderate	NR	c.3704 A > T	7	4–27 years	Homozygous	
				p.(Glu1235Val)				
Pakistani	Below −3 SD	Moderate to severe	Unable to read or write, could not speak simple phrases, and lack of self-care skills	c. 3243_3246delTCAG p.(Ser1081Argfs*8)	3	8–13 years	Homozygous	Gul et al.^10^
Iranian	Below −4 SD	Severe (IQ 60)	Facial dysmorphism (small ears, hypertelorism, notched nasal tip, and strabismus), joint stiffness (ankles), wheelchair bound, finger deformities, and seizure	c.2462 C > T p.(Thr821Met)	2	NA	Homozygous	Darvish et al.^20^
Southern Saudi Arabia	−7 SD	One member with intellectual disability	Seckel phenotype, intrauterine growth retardation, receding chin, high forehead, prominent nasal spine, hypoplastic alae nasi, low-set ears, 11 ribs, and steep acetabular roof	c. 3302-1 G > C	5	1–16 years	Homozygous	Al-Dosari et al.^11^
Pakistani	−8 to −12 SD	Yes	No obvious dysmorphic features, unable to speak, read, or write	c.18delC	3	7–18 years	Homozygous	Hussain et al.^8^
				p.(Ser7Profs*2)				
Pakistani	−10 to −12 SD	Yes	No obvious dysmorphic features	c.18delC	3	18–23 years	Homozygous	
				p.(Ser7Profs*2)				
Pakistani	−10 to −17 SD	Yes	No obvious dysmorphic features, friendly behavior, poor self-care skills, unable to read, and write	c.18delC p.(Ser7Profs*2)	4	10–30 years	Homozygous	
Turkish	NR	NR	Arthrogryposis: contractures of elbows, ulnar deviation of hands, talipes equinovarus; retromicrognathia, high-arched palate, crowded and decayed teeth, low-set ears, delayed bone age, abnormal wound healing, and normal growth parameters (no microcephaly)	c.763 A > G p.(Thr255Ala)	1	NA	Homozygous (reported as nonconsanguinity)	Bayram et al.^12^
Argentinian	Below −5 SD	Moderate to severe	Learning problems, height −1.63 SD (mean parental height +0.24 SD), corpus callosum hypoplasia, bilateral migration disorder with heterotopia of the Sylvian fissure and colpocephaly	c.289dupA (p. Thr97Asn*fs*Ter7) and c.1132 C > T (p.Arg378Ter)	1	20	Compound heterozygous	This work

Several genes are associated with autosomal recessive MCPH. In the majority of cases, the brain structure is normal, and the most frequent anomaly is a small brain with small gyri^1–4,13^. A simplified gyral pattern is frequent in patients with pathogenic variants in *ASPM*^3,4^, *WDR62*^14^ and *CIT*^3,4^. Patients with *WDR62* pathogenic variants show a wide spectrum of malformations, ranging from a small brain with a simplified gyral pattern to severe major brain structural malformations^3,4,14^. Cerebellar hypoplasia has been described in patients with *CDK5RAP2, ASPM, CENPE, SASS6, MFSD2A*, and *CIT* pathogenic variants^2,3,15^. Our patient has characteristics of typical MCPH: head circumference >3 SD below the population mean (−5.42 SD), and cerebral neuroimaging showing bilateral migration disorder with heterotopia in the absence of other concomitant findings.

*CENPJ* is a 17-exon gene mapping at 13q12.2, which encodes centromere protein J, or CENPJ, a protein localized to centrosomes in interphase and to the spindle poles during mitosis^3^. Cho et al. observed that the depletion of CENPJ protein disrupts centrosome integrity and that cells lacking CENPJ arrest in mitosis with multipolar spindles^16^. The centrosome plays a key role in regulating cell division, functioning as a microtubule organizing center. Bond et al. proposed that a centrosomal mechanism is responsible for determining brain size^9^. CENPJ interacts with proteins involved in MCPH, such as CEP135, CEP152, WDR62, ASPM, and STIL, and binds microtubules^17,18^. In recent years, the function of CENPJ in mice has been studied, confirming that this protein regulates progenitor division and neuronal migration in the cerebral cortex^19^.

In summary, our case represents a rather uncommon situation for the usual known context of MCPH and the *CENPJ* gene. These special characteristics include family origin (Argentinian), pedigree (nonconsanguineous), and genotype (a compound heterozygous case with two unreported variants predicting a truncated protein). NGS studies applied in a broader spectrum of clinical presentation of MCPH syndromes may discover additional patients and families with these characteristics and will help to provide accurate diagnosis, better phenotype delineation, and adequate genetic counseling.

## Data Availability

The relevant data from this Data Report are hosted at the Human Genome Variation database at 10.6084/m9.figshare.hgv.2858. 10.6084/m9.figshare.hgv.2861.
